# Confirmed diagnosis of classic Wiskott–Aldrich syndrome in East Africa: a case report

**DOI:** 10.1186/s13256-022-03517-1

**Published:** 2022-07-27

**Authors:** Mpokigwa Kiputa, Obrey Urio, Anna Maghembe, David Kombo, Sajda Dhalla, Victoria Ndembo, Kandi Muze, Mariam Kahwa, Zameer Fakih, Edward Kija

**Affiliations:** 1 Department of Paediatrics and Child Health, Muhimbili University of Health and Allied Sciences (MUHAS), Dar es Salaam, Tanzania; 2 Department of Paediatrics and Child Health, Muhimbili National Hospital, Dar es Salaam, Tanzania; 3 Department of Paediatrics and Child Health, Iringa Regional Hospital, Iringa, Tanzania; 4 Department of Paediatrics and Child Health, School of Medicine, MUHAS, 9 United Nations Road, Upanga West, P.O. Box 65001, Dar-es-salaam, Tanzania

**Keywords:** Micro thrombocytopenia, Eczema, Wiskott-Aldrich syndrome, Wiskott-Aldrich syndrome protein, Case report

## Abstract

**Introduction:**

Wiskott–Aldrich syndrome is a rare X-linked primary immunodeficiency that mostly presents with a classic triad of eczema, microthrombocytopenia, recurrent infections, and increased risk of autoimmunity/malignancies.

**Case presentation:**

We present an 8-month-old African male, born from nonconsanguineous parents and who presented with a history of eczematous skin rash since day 9 of life, with recurrent sinus infections, otitis media, and skin abscesses. An elder male sibling who had similar symptoms passed away during infancy. Investigations were consistent with microthrombocytopenia and significantly raised immunoglobulin E, while immunoglobulin A and immunoglobulin G were moderately elevated with normal immunoglobulin M. Genetic testing revealed the patient to be hemizygous for a pathogenic Wiskott–Aldrich syndrome gene variant (*NM_000377.2:c.403C>T*). He was managed conservatively with supportive treatment until he died a year later.

**Conclusion:**

Despite Wiskott–Aldrich syndrome being a rare disease, it should be considered as a differential in any male child who presents with microthrombocytopenia and recurrent infections, especially in low-resource settings where genetic testing is not routinely available.

## Introduction

Wiskott–Aldrich syndrome (WAS) is an inherited X-linked primary immunodeficiency disorder caused by mutations in the gene that encodes the Wiskott–Aldrich syndrome protein (WASP) [1]. The gene resides on short arm of chromosome Xp11.22–p11.23, its expression being limited to cells of non-erythroid hematopoietic lineage [2]. WASP regulates the assembly of actin filaments in the cytoskeleton [3].

The estimated incidence of WAS is 1 in 100,000 live male births, with no ethnic or geographical predilection [4, 5]. The average age at diagnosis for WAS is 2 years [6].

WAS has a wide range of clinical phenotypes that correlates with the mutation in the WAS gene. The spectrum ranges from less severe forms such as X-linked thrombocytopenia (XLT), characterized by low platelet count with absent or mild eczema/immunodeficiency, to a more severe form known as classic WAS, characterized by a triad of severe immunodeficiency, microthrombocytopenia, and eczema with an increased risk of developing autoimmunity and lymphoreticular tumors. Another phenotypic variation is X-linked neutropenia (XLN), associated with variable degrees of neutropenia [7, 8].

Genetic sequencing is required for a confirmatory diagnosis of WAS. Unfortunately it is not available in many low-resource settings, making clinical suspicion heavily relied upon. Early diagnosis and treatment prolong the life of patients with WAS [7]

The definitive management for WAS is with hematopoietic stem cell transplantation (HSCT), although gene therapy has also shown promising results [6, 9]. In our case report, we present a male infant from the Southern Highlands region of Tanzania who presented with the classic triad of WAS. This case is presented to highlight the challenges in diagnosis and management of WAS in a low-resource setting.

## Case presentation

An 8-month-old African male, born by spontaneous vaginal delivery from nonconsanguineous parents, was admitted at our hospital because of persistent eczematous skin rashes since day 9 of life. The rash started on the face, later progressing to involve the entire body except the palms and soles of the feet. The skin lesions were itchy with obvious peeling. He had significant improvement of the symptoms while using steroid creams, with flares upon cessation.

He also presented with a history of recurrent purulent nasal discharge with the first episode being at 4 months of age. Four episodes were reported since the onset of symptoms, which were at least a month apart, all being treated with a course of oral antibiotics.

Additionally, he had three episodes of bilateral purulent ear discharge starting at 4 months of age, which were associated with an on and off low-grade fever. Ever since, he had been experiencing multiple episodes of similar symptoms at least once a month.

There is a history of death of a male sibling at 8 months of age who had similar symptoms to the index patient. The only sibling who is alive is an 8-year-old female who is healthy with no similar symptoms. Furthermore, there is history of early pregnancy loss at 2 months of gestational age, but the sex of the abortus was not determined.

His mother received adequate prenatal care. He was delivered at term via caesarean section owing to abnormal lie with birth weight (BWT) of 3.5 kg with no obvious perinatal insult. After 72 hours they were discharged home with no reported complications.

Immunization was up to date; however, the Bacillus Calmette–Guérin (BCG) vaccine scar was not visible. Although he had weight faltering, his developmental milestones were appropriate for his age.

**Fig. 1:**
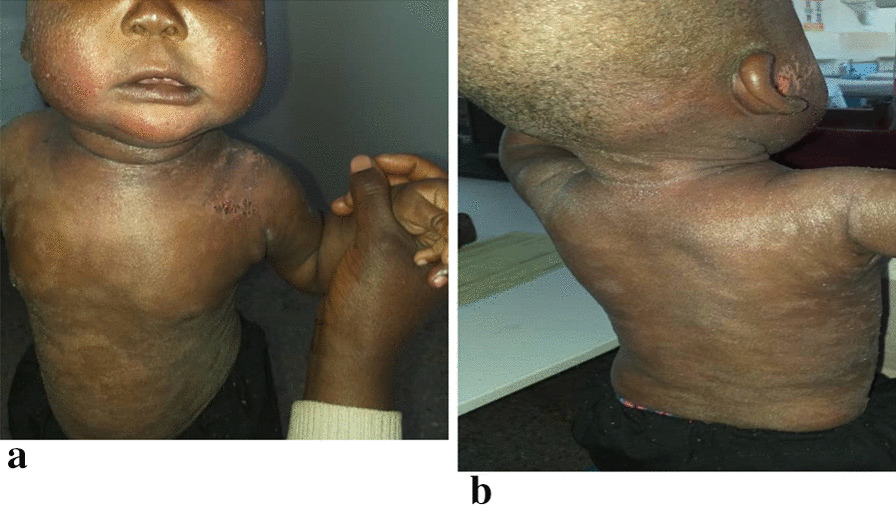
**a** Eczematous papules with erythema on the cheeks. Hypopigmented patches and excoriations on the trunk.** b** Crusted eczematous papules on the scalp with excoriated periauricular area

On examination, he had extensive confluence of hypopigmented patches with eczematous papules on the trunk, extremities, and scalp, as well as crusted plaques and excoriations on the periauricular area and skin folds. The lesions spared the palms, soles of the feet, and mucocutaneous membrane (Fig. 1a, b).

On otoscopic examination, there was purulent discharge visible on the external auditory meatus, with an intact keratinized tympanic membrane but with loss of the normal reflex.

Cardiovascular, respiratory, nervous ,and abdominal systems examination was normal.

**Fig. 2:**
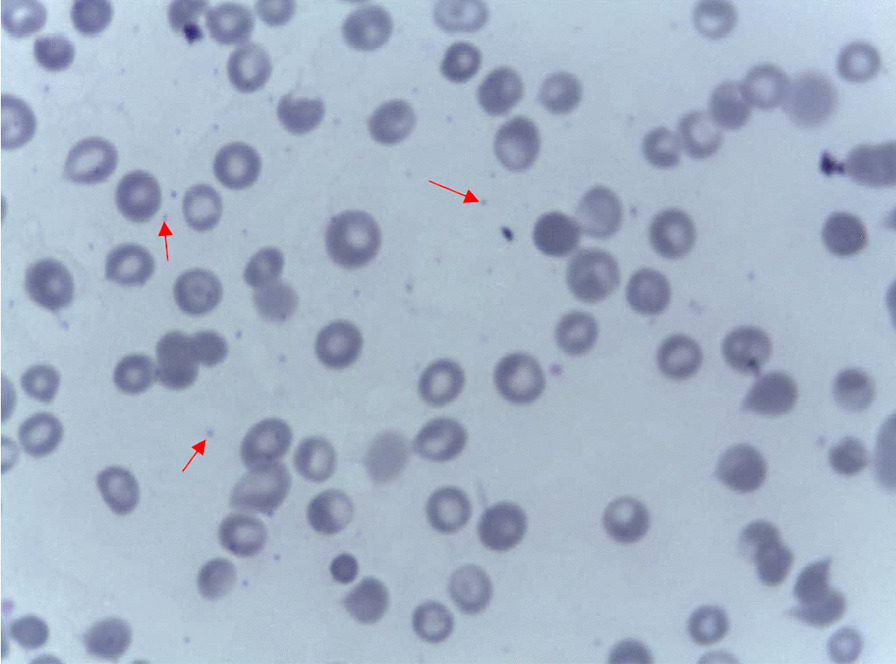
A Peripheral blood smear slide; Red arrows showing micro thrombocytes

Blood was taken for complete blood count (CBC), and the results showed mild normocytic normochromic anemia, low platelet count, and low mean platelet volume (MPV) (Table 1). A peripheral blood smear showed microthrombocytopenia (Fig. 2). HIV serological test was negative. Immunoglobulin assay revealed significantly elevated immunoglobulin E (IgE), elevated immunoglobulin A (IgA), and immunoglobulin G (IgG) with normal immunoglobulin M (IgM) (Table 2).

**Table 1:** CBC results

Hb	MCV	MCH	RDW	PLAT	WBC	NEUTR	MPV	LYM
9.28 g/dl	76.5 fl	25.7 pg	25.6%	70.9 k/µl	21.5 k/µl	2.13 kµ/l	3.62 fl	16.4 k/µl

*CBC* complete blood count,* Hb* haemoglobin,* MCV* mean corpuscular volume,* MCH* mean corpuscular haemoglobin,* RDW* red cell distribution width,* PLAT* platelets,* WBC* white blood cell count,* NEUTR* neutrophils count,* MPV* mean platelet volume,* LYM* lymphocytes count

**Table 2:** Other laboratory test results

IgG (172–1069 mg/dl)	IgE (0–230 IU/ml)	IgA (11–106 mg/dl)	IgM (33–126 mg/dl)	HIV-DNA PCR	LDH	DAT	Pus swab
1472	10,000	332	115	Negative	1313	Negative	No growth

*Ig* immunogloblin,* HIV* human immunodeficiency virus,* LDH* lactate dehydrogenase,* DAT* direct antiglobulin test

Given the clinical and laboratory presentation of our patient and his biological sex, we had a high index of suspicion of WAS. A dried blood spot was sent to Rostock, Germany, to test for WAS gene using CentoXone Solo [including next-generation sequencing (NGS)-based copy number variation (CNV) analysis]. A hemizygous pathogenic variant was identified in the WAS gene (*NM_000377.2:c.403C>T*). The WAS variant c.403C>T p.(Gln135*) creates a premature stop codon. Loss of function is the reported disease-associated mechanism for WAS.

### Management

The patient’s eczematous skin lesions were treated with hydrocortisone cream 1%. Mupirocin and clotrimazole creams were applied on skin folds. Antihistamine cream was given to curb severe itching.

Ear painting with cream (clotrimazole + gentamycin + betamethasone) was done on a weekly basis. The patient was discharged after a week with prophylactic oral cotrimoxazole 240 mg once daily (OD) and hematinics. Live attenuated vaccines (LAV), such as measles–rubella, were deferred for our patient.

### Clinical follow-up

Given the fact that our patient was coming from upcountry 330 miles away, we advised them to a monthly clinic in a nearby facility and to our clinic once every 3 months. In follow-up clinics, the patient’s platelet level kept decreasing gradually, but no bleeding tendencies were noticed initially. Hemoglobin levels remained low with high white cell count. The patient was maintained on prophylactic oral clotrimazole and hematinics.

In the days leading up to his death at a peripheral hospital, he presented with critically low levels of hemoglobin and thrombocytes. He had severe epistaxis and gum bleeding. He received multiple units of blood transfusion per body weight. He had torrential bleeding for several hours; however, platelet concentrates were not immediately available. Through verbal autopsy, we were able to establish the possible cause of death to be hypovolemic shock.

## Discussion

The findings of persistent eczema, recurrent ear infections, and microthrombocytopenia in our patient, plus a family history of an early death of a male sibling during infancy were more suggestive of WAS. Our patient had a WAS score of 4, which is consistent with classic WAS. The WAS scoring system is used to indicate disease severity and prognosis. A score of 1 indicates mild disease, while a score of 5 correlates with severe disease course and worst prognosis [10].

Genetic sequencing revealed the WAS variant c.403C>T p.(Gln 135*), which creates a premature stop codon resulting in loss of function for the WASP. It is classified as a pathogenic variant class 1. This confirmed the genetic diagnosis of X-linked Wiskott–Aldrich syndrome. We were unable to perform parental carrier testing to evaluate whether the variant is *de novo* or inherited, but with the history of another male sibling with similar symptoms dying in infancy, it is more suggestive of an X-linked recessive inheritance pattern.

This is a confirmed case of classic WAS from East Africa. Other previous cases reported in the scientific literature from Africa are by Lanzkowsky *et al*., which was published in 1965, of a child with a classic triad of WAS presenting at 1 month of age [11], and another case of classic WAS triad reported in Addis Ababa, Ethiopia by Deribssa *et al*., but it had no genetic confirmation [12]. Glanzmann *et al*. also reported a case of atypical WAS in a 3-month-old South African boy in 2020 [13]. The most recently reported case in East Africa was by Mawalla *et al*., in which the patient presented with recurrent fever, skin lesions, and bloody diarrhea with normal platelet size [14] (Table 3).

**Table 3:** Comparison of our case with other reported cases in Africa

Case report	Our case	Lanzkowsky *et al*. [11]	Deribssa* et al*. [12]	Glanzmann* et al*. [13]	Mawalla* et al*. [14]
Gender	Male	Male	Male	Male	Male
Family history	+	+	+	+	−
Consanguinity	−	−	−	−	
Thrombocytopenia	+	+	+	−	+
Small platelets	+		+	−	−
Eczema	+	+	+	+	+
Infections	+	+	+	+	+

The higher WAS score in our patient has been associated with an increased risk of malignancies and autoimmunity, as was shown by Sullivan *et al*. [6]. For that case, follow-up and monitoring was mandatory. Our patient died at a young age; he did not develop malignancy, as it tends to develop in later years with average age at onset of around 10 years [6].

Throughout follow-up, our patient was maintained on prophylactic antibiotic with cotrimoxazole, which has been shown to be effective in prevention of *Pneumocystis jirovecii* pneumonia [15, 16].

HSCT with a matched sibling donor or unrelated donor was indicated for our patient as an acceptable curative approach with desirable outcome [17–20]. Unfortunately, in our country, HSCT services are still under development, and currently only a small cohort of adults with multiple myeloma have received the service, with varied success rate. Gene therapy is also promising for the treatment of WAS [9].

## Conclusion

Despite its rarity, a high index of suspicion should be reserved for a male patient presenting with thrombocytopenia and recurrent infections. In a poor-resource setting like ours, a diagnosis of WAS can simply be overlooked. This case is presented as a reminder to health care service providers in Sub-Saharan Africa to be on the lookout for this debilitating form of combined immunodeficiency disease.

## Data Availability

Not applicable.
